# Human body donation and surgical training: a narrative review with global perspectives

**DOI:** 10.1007/s12565-022-00689-0

**Published:** 2022-10-13

**Authors:** Matthew J. Zdilla, Joy Y. Balta

**Affiliations:** 1 Department of Pathology, Anatomy, and Laboratory Medicine, West Virginia University School of Medicine, Morgantown, WV USA; 2 Anatomy Learning Institute, College of Health Sciences, Point Loma Nazarene University, San Diego, CA USA

**Keywords:** Anatomy, Anatomical education, Human body donation, Medical education, Surgery, Surgical education

## Abstract

Utilization of human material in surgical simulation training has been well-established as an effective teaching method. Despite the value of donor-based surgical simulation training, its application may be hampered by difficulties regarding access to donated bodies. Therefore, the aim of this review is to assess body donation and body acquisition practices with regard to surgical simulation training programs around the world. The results of this review highlight discrepancies regarding body donation practices and surgical simulation programs among continents and countries. The utilization of donor bodies in surgical simulation appears to mirror body donation practices. In countries that rely mostly or exclusively upon unclaimed bodies or executed criminals, there are scant reports of donor-based surgical simulation programs. In countries where willed-body donation is the principal source of human material, there tend to be many surgical simulation programs that incorporate human material as part of surgical training. This review suggests that, in anatomical and surgical education, the utilization of active willed-body donation programs, as opposed to the utilization of unclaimed human bodies, positively corresponds with the development of beneficial donor-based surgical simulation programs. Likewise, donor-based surgical simulation training programs may have an influence on the perpetualization of willed-body donations.

## Introduction

With regard to nomenclature throughout this review, the term *body donor(s)* will be used when human remains are known to have been donated. Otherwise, terms such as *deceased human bodies, deceased person,* or *decedents* will be used whenever possible. This nomenclature comes in an effort to limit or eliminate the commonly used term “cadaver” which objectifies the human body.

Deceased human bodies are utilized in many different ways as part of anatomical education and clinical training (Hammer et al. 2015; Hayashi et al. 2016; Homma et al. 2016, 2019; Salameh et al. 2020). Advancements in anatomical knowledge, research, surgical technology, surgical techniques, and pedagogy have, likewise, advanced donor-based surgical simulation (Gilbody et al. 2011; James et al. 2019; Shichinohe and Kobayashi 2021). This is still the case even though the use of animals is still very common and modern technology is becoming an integral part of surgical training (Robert et al. 2006). A recent review study highlighted the use of virtual reality and augmented reality in surgery and how they will soon become part of the standard of care (Ghaednia et al. 2021). Having said that, human remains are still considered high-fidelity with regard to training in several clinical disciplines, such as radiology (Balta et al. 2017), clinical skills (Byrne et al. 2008; Sharma et al. 2013), procedural skills (Kovacs et al. 2018), otolaryngology (Musbahi et al. 2017) and surgical skills (Hayashi et al. 2016) among others. Utilizing human material has been reported in several surgical specialties (James et al. 2019). Accordingly, donor-based surgical simulation has undoubtedly improved the practice of surgery and its outcomes. Thus, human body donors are foundational for such advancements. The value of working with body-donors significantly increase as medical schools reduce or eliminate the work with human body donors (Singal et al. 2020).

In 2012, the International Associations of Anatomists recommended that only donated bodies should be used in the study of anatomy. Despite the value of donor-based surgical simulation training, its application may be hampered by difficulties regarding access to donor bodies (Harris et al. 2015). This has opened the door to for-profit companies who would sell deceased human bodies that have been obtained under ethically questionable terms for the purpose of profit. In the United States, there is little regulatory guidance on the distribution and procurement of whole-body donors and, therefore, providing no processes for accountability and oversight (Champney 2016). When comparing organ to whole body donation, it becomes clear that people are more aware and willing to donate their organs versus their whole bodies (Oktem et al. 2020). More research has been conducted on the knowledge, attitude and practices regarding organ donation compared to whole body donation (Ballala et al. 2011). Indeed, many factors impact the access to donated human materials for education and research. These factors could be financial, legal, ethical, religious and technical (Balta et al. 2015). Accordingly, access to donor bodies and, thus, access to donor-based surgical simulation training, will differ based upon geographical location. To increase access to donors and maximize the benefits form this donation, recent studies have investigated different preservation methods that would increase the period by which clinicians can work with the donor while maintaining life like characteristics (Balta et al. 2015).

Therefore, the authors aim to detail body donation and body acquisition with regard to surgical simulation training programs around the world. It should be noted that this review does not detail each and every body donation program or donor-based surgical simulation program around the globe. Rather, the review aims to provide a thorough overview that accurately captures the essence of body donation and surgical training throughout the world.

## Africa

### Body donation and acquisition in Africa

Cultural practices in Africa have been thought to negatively impact body donation. As Akinola (2011) notes regarding the initiation of a formal body bequest program in Nigerian medical schools, “body donation is an alien concept to Africa.” There is some credence to the statement of Akinola (2011) due to the heavy reliance of unclaimed bodies throughout Africa. Many ethical questions have been raised about the use of unclaimed bodies (Jones and Whitaker 2012). Habicht et al. (2018) documented that, of 14 African countries with available information, eight use only unclaimed bodies (Ethiopia, Ivory Coast, Nigeria—also use bodies of executed criminals, Rwanda, Senegal, Tanzania, Uganda, Zambia), while four rely mostly on unclaimed bodies (Ghana, Kenya, Malawi, Zimbabwe), just one utilized mostly donor bodies (South Africa), and one, Libya, use “other sources.” Indeed, reports have noted that the only source of bodies in Libya is from importation from India (Gangata et al. 2010; Habicht et al. 2018).

Thus, most African countries continue to rely on unclaimed bodies for dissection programs or, otherwise, bequests from white Africans according to De Gamma et al. (2020). Indeed, Kramer and Hutchinson (2015) report that, in South Africa, black Africans are less willing than others to donate their bodies due perhaps to cultural beliefs and the country’s political history. A qualitative study recently assessed the cultural practices of the Zulu ethnic group with regard to notions of the body and body donation (De Gamma et al. 2020). The study identified themes which suggested that body donation is unlikely to be considered an acceptable practice among Zulus due to need to preserve the linkage between the physical human body and the spirit of the deceased person, and the perceived ongoing relationship between the spirit of the dead and the living (De Gamma et al. 2020).

### Surgical training in Africa

Perhaps partly due to a paucity of donor bodies, there are few reports regarding the use of human materials in surgical simulation in Africa. That stated, there are reports of African surgeons taking part in such surgical simulation training outside of Africa (Blázquez Hernando et al. 2020). While establishing local body donation programs could help increase access to donors, and, therefore, provide opportunities for surgical simulation. The main obstacles from achieving such goals would be some African cultural and spiritual practices. An assessment of donor-based surgical simulation with regard to body donation practices in Africa highlights the importance of international training programs utilizing similar models.

## Asia

### Body donation and acquisition in Asia

Asia is a remarkably culturally diverse continent, spanning from Japan to China, India, Iran all the way to Lebanon and Turkey. Over the years, several articles have been published about the limited access to deceased human bodies in mainland China (Rokade and Gaikawad 2012; Zhang et al. 2014; Chen et al. 2018). Some of the reasons behind the paucity of donation include the influence of personal spiritual beliefs and logistical reasons, such as legislations and lack of effective means of donation (Zhang et al. 2014). Suggestions have been made on effective ways to alleviate the shortage of donors such as promoting body donation on national levels along with developing humane and efficient protocols for body donation (Chen et al. 2018). Similarly, medical colleges in India are not able to meet the demand of human body donors for their increasing number of medical schools (Rokade and Gaikawad 2012). One of the primary reasons for the shortage of body donation is the lack of awareness regarding the option to donate one’s body (Rokade and Gaikawad 2012).

In addition to China and India, many other Asian countries such as Bangladesh, Indonesia, Iran and Turkey seem to have similar challenges (Habicht et al. 2018). Other Asian countries rely solely on unclaimed bodies, such as Bahrain, Jordan, Oman, Qatar, United Arab Emirates, and Saudi Arabia (Habicht et al. 2018). Cultural and religious reasons could be behind the lack of body donation programs in these countries (Habbal 2009). This has led to the import of decedents into these countries to meet the demand for anatomical and medical education.

On the other hand, a small number of Asian countries such as Japan, Korea and Sri Lanka have had success in meeting the demand for donor bodies through their body donation programs (Habicht et al. 2018). Some of this success could be attributed to the strict regulations and the care to maintain respect for the donors. Indeed, in 2008, Japan experienced 210,000 persons registered for donation, a number which has been described as generally sufficient for the need of academic institutions in Japan (Sakai 2008). Some religious beliefs encourage body donation as an act of compassion and charity described as “dana” by Buddhists who follow Theravada Buddhism. Being the predominant belief in Sri Lanka, this has led to an excess in receiving donated human bodies and, in some instances, the refusal of some donations due to an overabundance (Subasinghe and Jones 2015).

The “silent mentor” program originated from Tzu Chi University and has been espoused in other universities in Asia including, for example, the University of Malaya, the National University of Singapore, and institutions in Hong Kong and Myanmar (Santibanez et al. 2016; Saw 2018).

### Surgical training in Asia

In Turkey, the Surgical Anatomy and Technologies Association (SATA) organized a panel titled “Ethical and Proper Use of Cadavers in Surgical Education” during the First National Anatomy and Cadaveric Dissection Symposium of SATA, held in the year 2018 (Selcuk et al. 2019). Decisions emerging from SATA will be useful to other programs that are utilizing donor-based surgical simulation and those programs that plan to endeavor in similar training (Selcuk et al. 2019). Aside from reporting the ethical and proper use recommendations of SATA, a study by Selcuk et al. (2019) noted that if donor bodies are utilized effectively and properly, courses will produce successful educational results in a cost-effective manner.

A recent and comprehensive systematic review of decedent-based simulation for surgical training reported only one study from the continent of Asia, specifically from the country of Japan (James et al. 2019). While the exclusion factors (measuring the educational impact) in the aforementioned study could be the reason behind the limited findings, limited access to human bodies in certain parts of Asia could also play a role in the few studies published from this region.

Regarding laparoscopic procedures, the Clinical Skills Training Center within the Department of anatomy at Seth G S Medical college and K E M Hospital in Mumbai, India noted success in training laparoscopic techniques which included cholecystectomy, appendectomy, splenectomy, intestinal explorations, mesenteric lymph node biopsy and varicocele-vein occlusion utilizing unembalmed human bodies (Supe et al. 2005). Likewise, Chulalongkorn University in Thailand, successfully piloted the so-called Minimally Invasive Surgery Training in Soft preserved body donors, utilizing laparoscopic surgery in upper gastrointestinal, colorectal, hepatopancreaticobiliary, and solid organ surgeries including laparoscopic proctocolectomy (Udomsawaengsup et al. 2005; Pattana-arun et al. 2005).

With regard to neurosurgery, Suri et al. (2014) described practical guidelines for creating a donor-based neurosurgery skills training laboratory in India. Table 1 provides a summary of the dominating scene in each continent with information on the sources of deceased human bodies, availability of donation programs, acceptance of body donation, work with body donors in medical education and surgical training.

**Table 1:** Summary from each continent on the sources of deceased human bodies, availability of donation programs, acceptance of body donation, work with body donors in medical education and surgical training

Continent	Source of bodies	Donation programs	Acceptance of body donation	Donors in Med education	Donors in surgical training
Africa	Unclaimed bodies	Rarely available	Not common	Yes	No
Asia	Mostly unclaimed bodies	Mostly unavailable^a^	Depends on culture	Yes	Yes
Australia/Oceania	Body donation	Available	Common	Yes	Yes
Europe	Body donation	Available	Common	Yes	Yes
North American	Body donation	Available	Common	Yes	Yes
South America	Mostly unclaimed bodies	Mostly unavailable	Not common	Yes	No

^a^Varies highly in different parts of Asia. While many countries rely on unclaimed bodies, Japan, Korea and Sri Lanka have had success in meeting the demand for donor bodies through their body donation programs

## Australia/Oceania

### *Body donation and acquisition in Australia/*Oceania

Body donation programs have been described as the major or exclusive source of deceased human bodies in Australia (Alexander et al. 2014; Habicht et al. 2018) and New Zealand (Cornwall et al. 2012; Biasutto et al. 2014; Habicht et al. 2018). From examination of varied university websites, there seems to be 13 body donation programs Australia and two in New Zealand. It is unclear from the existing literature on whether the current number of donations meets the demand.

However, medical schools in at least three countries in Oceana, namely, Fiji, Samoa, and Solomon Islands, do not use anatomical dissection at all (Habicht et al. 2018). Whether the non-existent use of anatomical dissection is by choice or by necessity is unclear from the literature.

### Surgical training in Australia/Oceania

In Australia, the Clinical Training and Evaluation Centre at the University of Western Australia is an active site for educational innovations which include the Advanced Anatomy of Exposure course, entailing unembalmed body donor workshops for advanced vascular surgical training (Jansen et al. 2014), as well as hosting 26 General Surgery Core Skills Workshops between 2007 and 2019 (Chai et al. 2019). Indeed, Chai et al. (2019) note that “[f]resh frozen cadaver workshops are of value in the face of current surgical training challenges in providing an efficient, effective and safe environment.”

At James Cook University in Cairns, Australia, a 3-day donor-based simulation program known as the “Anatomy of Surgical Exposure” has been implemented to teach a whole-body course of operative general surgery in a region-by-region approach to both Australian and New Zealand residents (Killoran et al. 2021). Killoran et al. (2021) utilized a formal Objective Structured Assessment of Technical Skill tool to assess residents in the Anatomy of Surgical Exposure course. The results of Killoran et al. (2021) indicated that the 3-day course did not significantly improve technical skill, but technical skill was significantly correlated with postgraduate year of education.

Based at the medical school of the University of New England in Amidale, Australia, a rural donor-based neurosurgical laboratory had been established to provide hands-on training in the areas of neurosurgery and spine surgery (Smith et al. 2015). Bodies and surgical equipment were sourced from various veterinary practices and commercial companies (Smith et al. 2015). Using human and animal remains, this laboratory provided trainees with hands-on opportunities to improve their surgical skills, neuroanatomical knowledge, and familiarity with highly specialized surgical equipment (Smith et al. 2015).

In general, reports of donor-based surgical training primarily arise from well-developed countries. Thus, Smith et al. (2015) provide evidence for the feasibility of cadaver-based surgical training in remote locations. Furthermore, it is important to note that the creation of a human body donor laboratory that includes strong downdraft ventilation can be accomplished with little funding and with readily available materials (Zdilla 2020, 2021a; b).

## Europe

### Body donation and acquisition in Europe

Physicians from European countries have made a significant contribution to the discipline of anatomy and were pioneers in working with deceased human bodies. For example, the golden age of Greece, housed the beginning of the intellectual development of anatomy (Phillips 1973). Other scientists, such as Alcmaeon and Empedocles from Italy, left their mark on anatomy by being the first to dissect animal cadavers (Malomo et al. 2006). Andreas Vesalius has been described as the reformer of anatomy as he challenged his teachers and the concepts written by Galenus (Silverman 1991; Gregory and Cole 2002). In 1539, Vesalius published views on bloodletting in the “Venesection letter” and started working on the magnum opus, *De Humani Corporis Fabrica* (On the Structure of the Human Body) (Silverman 1991).

While the teachings of Vesalius encouraged the dissection of deceased human bodies, the only legal supply of subjects was from bodies of murderers who were sentenced to be hanged and dissected (Harris 1920). This source was too limited for the needs of medical schools and, in some cases, led to illegal methods of obtaining bodies such as bodysnatching or murder until the legislation of the Anatomy Act in 1832 (Mitchell et al. 2011). The new act allowed the dissection of bodies, from individuals who died in public institutions and were unclaimed by their relatives, by students under the supervision of a properly licensed teacher (Harris 1920).

Nowadays, body donation is extremely regulated in European countries and is becoming more popular in countries like the Netherlands, where body donation programs had to stop registering donors in 2012 to prevent the surplus of bodies (Bolt et al. 2012). The University of Bologna in Italy has also experienced increased donor enrollment and, as a result, improved teaching and training quality in recent years (Orsini 2021). Conversely, some European countries such as Greece are still facing low rates of body donation (Halou et al. 2013). Halou et al. (2013) noted that, among populations from five major Greek cities, willingness of body donation was influenced by educational level, annual family income, employment status, presence of comorbidities, and religious beliefs. While certain religious beliefs in Greece may contribute to decreased willed-body donation, faculty of the Medical University of Silesia in Katowice, Poland report that most donors in their Conscious Body Donation Program are Catholic (Bajor et al. 2015). Thus, varied religious beliefs may influence body donation in varied ways.

Changes in regulations have also had an impact on body donation and how donors can be utilized in research and education. In the United Kingdom, changes in the Human Tissue Act which impacts England, Wales, and Northern Ireland along with the Anatomy Act which governs Scotland, have made it possible to conduct surgical procedures on human body donors (Pryde and Black 2005; Taylor and Wilson 2007). These changes led to an increased investment in the development of donor-based training programs in the United Kingdom.

In 2008 the Trans-European Pedagogic and Anatomic Research Group (TEPARG) gave its summary of body donation throughout Europe (McHanwell et al. 2008). Likewise, updates regarding the legal and ethical framework regarding body donation in Europe have provided perspectives from varied individuals representing Austria, France, Germany, Italy, Malta, the Netherlands, Portugal, Romania, Spain, Switzerland, Turkey, and the United Kingdom (Riederer et al. 2012).

### Surgical training in Europe

In Germany, Back et al. (2017) had reported successful integration of a donor-based Advanced Surgical Skills for Exposure in Trauma course as part of the 5-day German Bundeswehr Medical Service’s War Surgery Course. Prior to the incorporation of deceased human bodies, the program used a porcine model; now, the porcine and human models are both used (Back et al. 2017). Indeed, other training programs, like those in ophthalmology, have utilized porcine models (or models using other species) alongside body donors (Pujari et al. 2021).

Also from Germany, Lorenz et al. (2017) describe the German Hernia School, which entails work with unembalmed deceased human bodies as a component of the standardized curriculum for continuing training in hernia surgery.

Postgraduate specialty trainees in obstetrics and gynecology at the Newcastle Surgical Training Centre in England (licensed under the Human Tissue Authority) were introduced to a donor-based surgery program (Lim et al. 2018). The participants in the program rated a consistent improvement in confidence and found the program to be a positive experience (Lim et al. 2018). In addition, in the United Kingdom, a body donor laboratory, housed within a tertiary referral hospital, has served as a site for successful hands-on training courses (~ 60 courses per year) across a wide range of disciplines (Holland et al. 2011).

Throughout the United Kingdom, donor-based surgical simulation courses, such as the Royal college of Surgeons Definitive Surgical Trauma skills course, are commonly viewed as highly desirable or essential learning (Hughes et al. 2019). Such training, like that of the Urology Human Cadaver Training Program developed by the British Association of Urological Surgeons was found to have a high value for educational influence and cost-effectiveness (Ahmed et al. 2015). Other studies emerging from the United Kingdom have, likewise, noted the overall effectiveness as well as the cost-effectiveness of donor-based surgical simulation such as that regarding reconstructive microsurgery training by Chouari et al. (2018).

A group of plastic surgeons in Switzerland have emphasized the important role of donor-based surgical simulation training for new and highly technical procedures and, particularly, that there is a need for donor-based training due to patient anatomical variability (Krähenbühl et al. 2017). Likewise, similar sentiments have been echoed across Europe (Delpech et al. 2017; Chouari et al. 2018). Indeed, donor-based surgical simulation has been perceived by surgeons practicing in the UK to be a significantly overall better model for laparoscopic training than high-fidelity virtual reality simulator training regardless of the complexity of the operative procedure being performed (Sharma and Horgan 2012).

## North America

### Body donation and acquisition in North America

Like many other parts of the world, North America has had a muddy history regarding the acquisition of deceased human bodies. The introduction of laws such as the Uniform Anatomical Gift Act in the United States of America in 1968 helped improve some of the ambiguity with previous practices based on the anatomy acts (Sadler et al. 1968). Efforts continue to be made by legislators to protect whole body donors, such as the recent Consensual Donation and Research Integrity Act (Consensual Donation and Research Integrity Act 2021). Likewise, many voices from the academic community advocate for the legal oversight of body acquisition being facilitated by for-profit companies. Many questions are raised around the ethical practices of the aforementioned companies, especially with regard to the buying and selling of deceased human bodies without oversight (Champney 2016). A study compared the differences between a body donation program verses a for for-profit company and concluded donors to that company were significantly younger, more likely to be form a minority group and likely to have died from cancer (Anteby and Hyman 2008). These businesses continue to grow and expand in the United States as body donation becomes more common. Currently, there are over 134 body donation programs operating in the US with new programs continuingly being developed (Zealley et al. 2022).

According to survey responses from Habicht et al. (2018), acquisition of bodies in Canada is through exclusively body donation; in Mexico, acquisition of bodies is from mostly unclaimed bodies; in Nicaragua, acquisition of bodies is from exclusively unclaimed bodies.

### Surgical training in North America

With an appreciation that there is widely available access to donor bodies in the United States, it is of little surprise that several studies have reported the use of body donors in surgical training. Likewise, the United States has myriad colleges, universities, and hospitals that serve to train surgeons from across the world. Thus, donor-based surgical training in the United States of particular global interest, akin to other programs that provide similar training to international trainees, to be discussed subsequently (Blázquez Hernando et al. 2020; Killoran et al. 2021).

There have been several reports of utilizing human materials for trauma surgical training in the United States. For example, The Eastern Association for the Surgery of Trauma (USA-based) utilized donor-based surgical training via filming procedures performed on body donors and subsequently distributing footage as a master class video presentation (Warriner et al. 2020). Other, approaches to surgical training include that of the collaborative efforts of West Virginia University (WVU), the Navy Trauma Training Center and the LAC + USC Medical Center in Los Angeles, using perfused donor models for the management of cardiovascular injury as a component of combat trauma surgical training (Grabo et al. 2020). The forward surgical teams from the Israeli Defense Forces (consisting of junior and experienced surgeons, anesthesiologists, and surgical scrub technicians) attended the 4-day combat surgical skills course and demonstrated improvements in both team dynamics and technical skills evaluations (Grabo et al. 2020).

In addition to combat trauma training, perfused body donors have been utilized for realistic simulation models like the so-called “mock operating room” for neurosurgical procedures at the University of Southern California (USC) (Zada et al. 2018). In addition, at USC, it was determined that implementation of a cardiac surgery simulation curriculum utilizing an unembalmed tissue donor laboratory is feasible as evidenced by a pilot among residents that demonstrated particularly favorable results (Baker et al. 2012). Likewise, USC plastic surgery residents noted improved technique, speed, safety, and anatomical knowledge after undergoing a unembalmed tissue-based preoperative surgical rehearsal program (Weber et al. 2017).

The Food and Drug Administration of the United States approved the robotic surgery platform for use in gynecologic surgery in 2005 (Boitano et al. 2021). Hence, the Society of Gynecologic Oncology developed a robotic surgery course in a “robotic cadaver lab” among gynecologic oncology fellows and attendings (Boitano et al. 2021). Likewise, with regard to urology, a pilot study at WVU demonstrated the utility of perfused body donor simulation in open and endoscopic surgical procedures in the training of urology residents in the WVU Fresh Tissue Training Program (McClelland et al. 2021). In addition, obstetrics and gynecology residents at the University of Pittsburgh, Magee-Women’s Hospital underwent a pilot training session to teach the retropubic midurethral sling, proctored by members of the Urogynecology Division of the hospital, which blended a brief lecture with models and donor simulation with positive results (Oliphant et al. 2017).

A survey of 229 American Association of Colleges of Podiatric Medicine-approved residency programs noted that the most reported type of donor labs available were medical company-sponsored and hospital-sponsored (Chu et al. 2020). Overall, 87.9% of the surveyed residents found that body donor lab is either extremely beneficial (57.8%) or somewhat beneficial (30.1%). The most important factors perceived in a successful donor-based lab were faculty instruction, accessibility of the lab, and the availability of instrumentation/hardware (Chu et al. 2020).

The Department of Ophthalmology at Queen’s University in Ontario, Canada produced a novel donor-based surgical simulation, whereby rust rings were created on donors’ eyes, the eyes were mounted on suction plates at slit lamps and medical students, ophthalmology residents, and faculty practiced rust ring removal—an exercise which was determined to be a valid training tool that could be used by trainees to gain comfort with the procedure before removal on live patients (Mednick et al. 2017).

## South America

### Body donation and acquisition in South America

In the year 2012, the International Federation of Associations of Anatomists recommended that only donated bodies be used in the study of anatomy. Though, as evidenced by this report, many countries continue to depend mainly or exclusively on unclaimed bodies. Likewise, the number of unclaimed bodies may be inadequate without the assistance of willing donors.

The Federal University of Health Sciences of Porto Alegre, Brazil (UFCSPA) has demonstrated that, by implementing a body donation program, a curriculum reliant upon of mostly unclaimed bodies (i.e., UFCSPA) can transition to a curriculum, where most of the bodies are sourced from willing donors (da Rocha et al. 2013). Furthermore, as a consequence of implementation of a body donor program, da Rocha et al. (2013) describes both an increase in both the quality and quantity of human anatomical material available for educational purposes.

Since the early 1990s, Chile has experienced a many-fold increase in the number of medical schools. The newest medical schools in Chile rely upon artificial models and the older medical schools rely upon long-standing anatomic specimens from museum collections (Gatica-Araneda and Alfaro-Toloza 2014). As Gatica-Araneda and Alfaro-Toloza (2014) note, only one Chilean university (i.e., Universidad de Chile) has a body donation program.

The National University of Cordoba in Argentina, only receives donated bodies; however, limited donations have resulted in a critical shortage that has placed both education and research at risk (Biasutto et al. 2021). Biasutto et al. (2021) surveyed thousands of individuals living in Cordoba and identified that cultural, religious, and inadequate public information might be implicated in the shortage of donors.

### Surgical training in South America

Like the African continent, South America has a paucity of reported donor-based surgical simulation programs. However, the reasons for the limited donor-based surgical simulation programs, reported in the literature, between Africa and South America appear to be due to different reasons. South American medical schools mainly rely on museum specimens or new models; however, a few have donor body programs including for example Universidad de Chile, UFCSPA, and National University of Cordoba. Thus, several schools in South America have the capability to develop surgical simulation programs, whereas others that do not have donor programs in place would have more significant obstacles with regard to instituting donor-based surgical simulation. Some schools, like the National University of Cordoba receive donated bodies; however, the donated bodies are in limited number which might make donor-based surgical simulation training particularly difficult or not feasible without an increase in donors. However, increasing body donation is possible by increased outreach and public education—strategies that have increased body donations elsewhere (Santibanez et al. 2016).

## Intercontinental

### Intercontinental body donation and acquisition

As aforementioned, there is a wide spectrum of needs, policies, regulations, experiences, and challenges across the above-mentioned six continents. This necessitates the need for global collaborations among body donation programs and individuals working with human body donors. Several groups such as the International Federation of Associations of Anatomists-Ethics and Medical Humanities and the American Association for Anatomy-Human Body Donor Task Force are working in these directions and more is needed to advocate for the donors’ best interests and maximize the benefits from this generous gift. These efforts are important to help share experiences and promote best practices.

On the other hand, questions could be raised on the possibility of establishing an intercontinental body donation database to help meet the demand in countries facing low rates of body donation. These suggestions have always been faced with ethical, legal and financial challenges. Some of these ethical questions are related to the donors’ knowledge of such activities and their ability to choose which countries they would be willing to donate to. Moreover, it is difficult to oversee the work being done with the donors and, likewise, ensure proper respect for donor bodies. Moreover, the legal frameworks governing the work with deceased human bodies might impact the ability to establish such collaborations.

While this formal intercontinental collaboration remains to be a suggested solution, many entities are already sending or receiving deceased human bodies across international borders. Questions are raised about the ethics of such practice. As a relatively expensive option, it is important to highlight the notion that monetary profits may be gained as a result of body donation. The concept of profit neutrality associated with body donation is particularly important to consider, especially in these circumstances. These practices can also create new challenges for example, donors are being sent from India to other countries, while local medical colleges in India are not able to meet the demand for their own institutions.

### Intercontinental surgical training

There are several donor-based surgical simulation courses, like the aforementioned “Anatomy of Surgical Exposure” course, that are inclusive of an international population. For example, Blázquez Hernando et al. (2020) report an assessment of the utility of a hands-on workshop on abdominal wall reconstruction, which has been ongoing for the past 6 years. The course, based at Henares University Hospital, which has an educational collaboration program with Francisco de Vitoria University, Spain, is a 2-day long course with 10–12 experienced surgeons in attendance for the benefit of the trainees, numbering 192, who come from Europe, South Africa, and the Middle East (Blázquez Hernando et al. 2020). The trainee–surgeons reported very-high satisfaction with the donor-based simulation (Blázquez Hernando et al. 2020).

Conversely, rather than surgeons traveling internationally, the donor-based surgical training module can travel internationally. For example, Aboud et al. (2015) reported a “Live Cadaver Model” in which artificial blood was pumped through heads with artificial and real aneurysms for surgical training at different locations. Aboud et al. (2015) report training 203 neurosurgical residents and 89 neurosurgeons and faculty members among 13 courses held in the United States and abroad (e.g., National Yang-Ming University, Taipei, Taiwan). Similarly, many surgical training models have utilized perfused human body donors (Willaert et al. 2018). Such models include perfusion of saline mimicking natural cerebrospinal fluid flow for the surgical simulation of neuro-endoscopic procedures (Winer et al. 2015).

It is worth mentioning that the language barrier is one of the main limitations of this review. While many of the university websites provided English translations, it was difficult to gather information from websites that did not offer this service. Therefore, the authors were biased in relying on sources written in English.

## Conclusions

The results of this review highlight discrepancies regarding body donation practices between continents and countries. Regardless of international recommendations for best practices, many countries continue to rely upon mostly or exclusively unclaimed bodies. The practice of donor-based surgical simulation as well as reports of novel utilization of donor bodies in surgical simulation appear to mirror body donation practices. In countries that rely mostly or exclusively upon unclaimed bodies and executed criminals, there are scant reports of decedent-based surgical simulation programs. In countries where willed-body donation is common, there tend to be many donor-based surgical simulation programs. Moreover, nearly all studies of donor-based surgical simulation programs note that the programs are beneficial and effective. Likewise, several reports have made note of the cost-effectiveness of donor-based simulation training.

Difficulties in developing a vibrant willed-body donation program are diverse and differ among varied cultures and geography. However, this review suggests that the lack of surgical simulation programs (programs which reports overwhelmingly suggest are efficacious) might be reflective of difficulties in developing a vibrant and active willed-body donation program. Thus, medical schools might strive to develop active willed-body donation programs to subsequently incorporate beneficial donor-based surgical simulation programs. Such surgical simulation programs may, in effect, aid in the perpetualization of willed-donor programs. This report also highlights the important contribution and impact that that body donors have upon surgical trainees and, as an extension, patient care.
